# Supplementary material for: Efficient soft-chemical synthesis of large van-der-Waals crystals of the room-temperature ferromagnet 1T-CrTe2
Source: J Mater Chem A Mater. 2024 Dec 10;13(21):15798–809. doi: 10.1039/d4ta05649c (PMC11629937; doi:10.1039/d4ta05649c)
Supplement: TA-013-D4TA05649C-s001 [file TA-013-D4TA05649C-s001.pdf]

Supplementary Information (SI) for Journal of Materials Chemistry A.
This journal is © The Royal Society of Chemistry 2024

Supplementary Information – Efficient Soft-Chemical Synthesis of Large van-der-Waals Crystals of the Room-Temperature Ferromagnet 1T-$CrTe_2$

Kai Daniel Röseler,[†] Catherine Witteveen,[†] Céline Besnard,[†] Vladimir Pomjakushin,[‡] Harald O. Jeschke,[¶] and Fabian O. von Rohr[*,†]

†*Department of Quantum Matter Physics, University of Geneva, CH-1211 Geneva, Switzerland*

‡*Laboratory for Neutron Scattering and Imaging, Paul Scherrer Institute, CH-5232 Villigen PSI, Switzerland*

¶*Research Institute for Interdisciplinary Science, Okayama University, Okayama 700-8530, Japan*

E-mail: fabian.vonrohr@unige.ch

Supporting Information Available

Photographs of 1T-$CrTe_2$ crystals using diluted acids

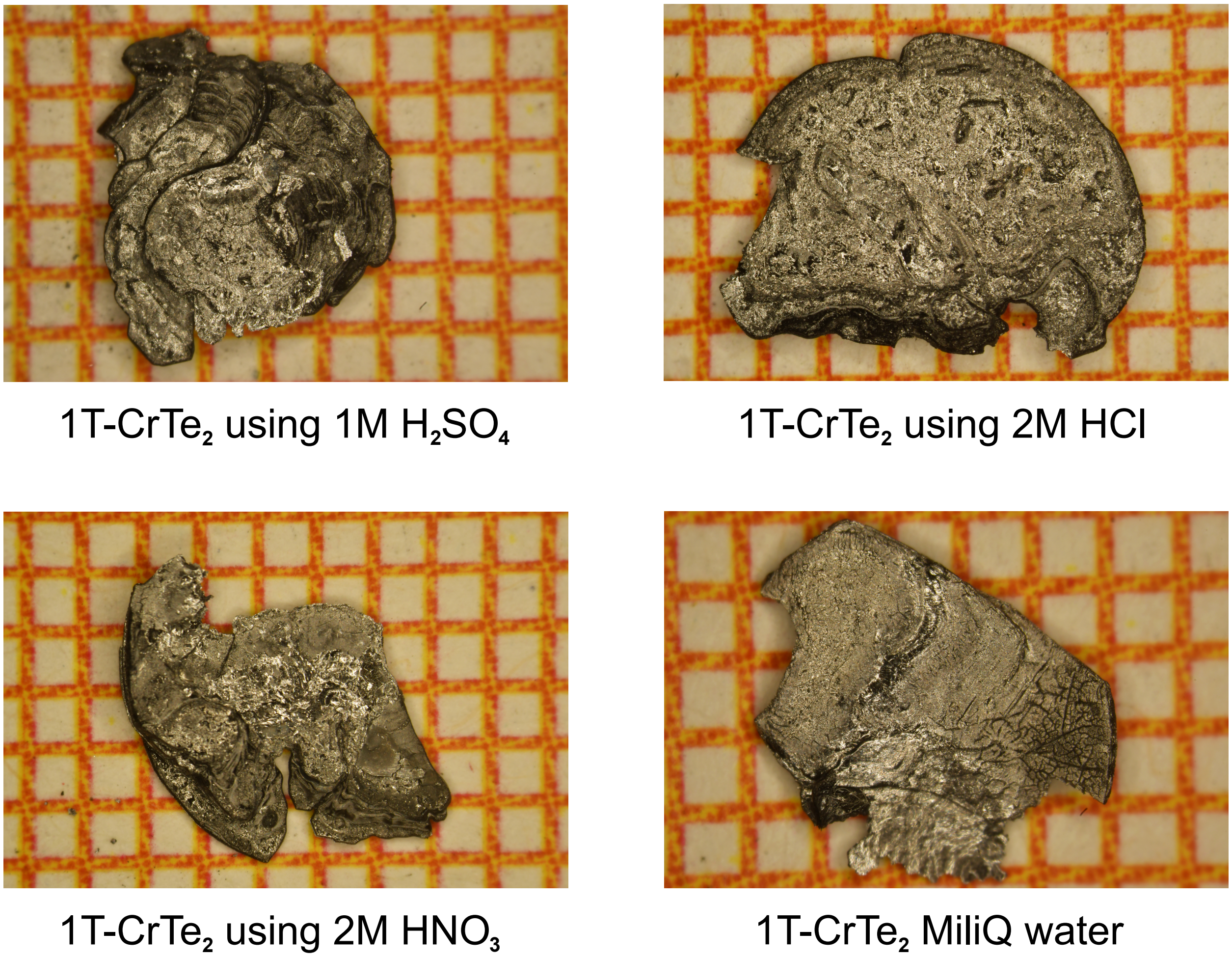

Figure 1: Photographs of 1T-$CrTe_2$ crystals obtained by the deintercalation of flux-grown $LiCrTe_2$ with diluted H_2SO_4, HCl, HNO_3 and Mili-Q water. All crystals were placed on millimeter spaced graph paper.

Reconstruction diffraction planes of SXRD measurements

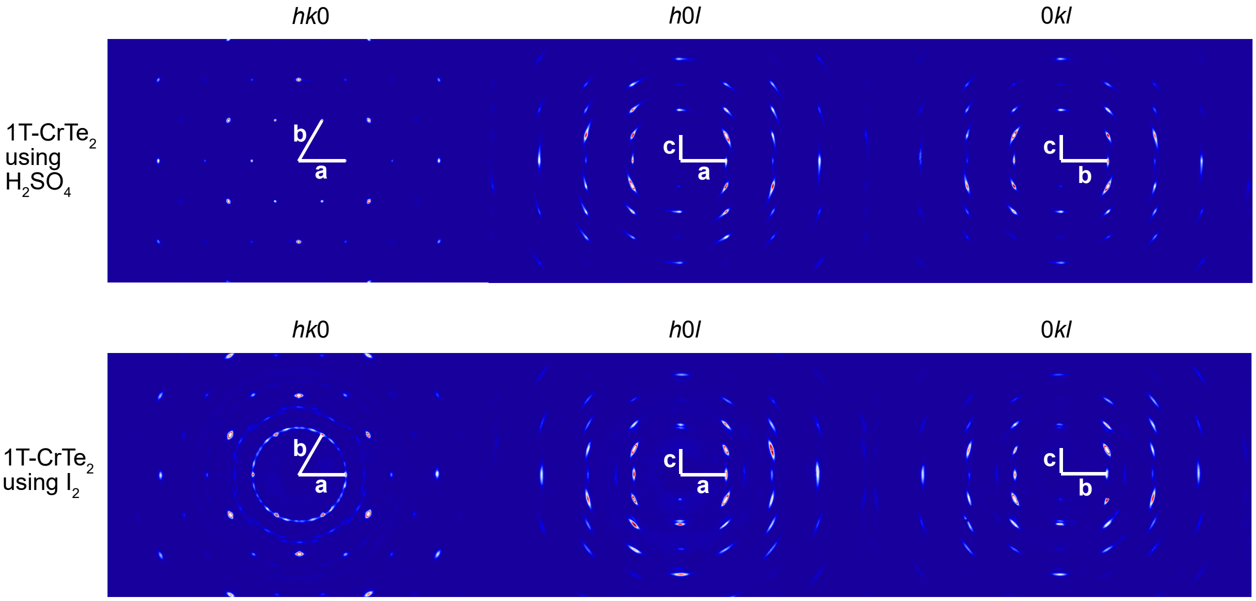

Figure 2: Reconstruction of the $hk0$, $h0l$ and $0kl$ planes from the SXRD data of 1T-$CrTe_2$ synthesized by deintercalation using diluted H_2SO_4 and I_2 in acetonitrile.

Pseudo exponential fit for deintercalation using I_2

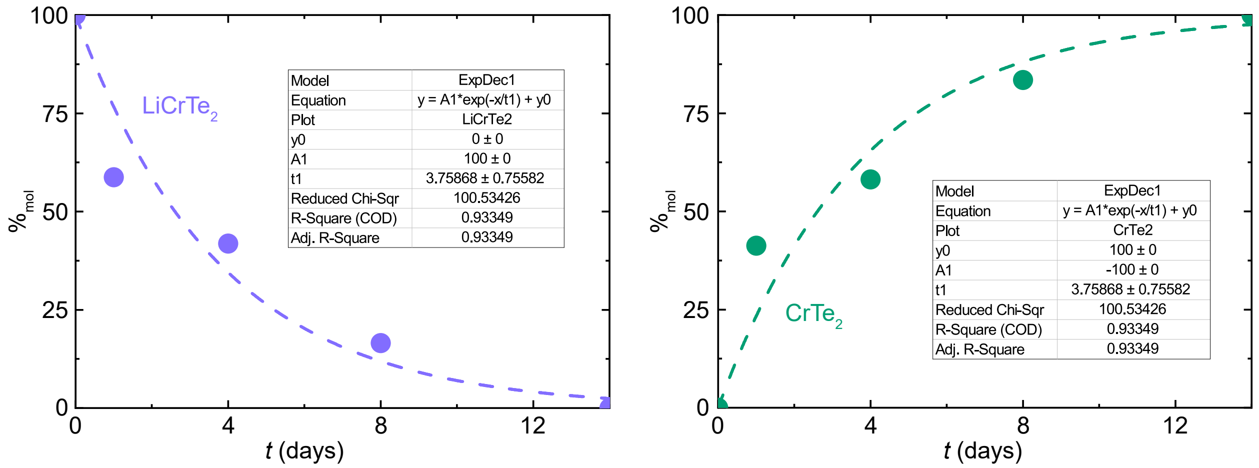

Figure 3: Pseudo exponential fits to show the general trend of molar percentages of $LiCrTe_2$ and 1T-$CrTe_2$ determined by refined PXRD patterns ob $LiCrTe_2$ deintercalated using 0.04 M solution of I_2 in acetonitrile. Crystals were deintercalated for 1, 4, 8 and 14 days respectively. For simplicity, Te was excluded and the sum of weight percentage of $LiCrTe_2$ and 1T-$CrTe_2$ scaled up to 100%.

Magnetic data of 1T-$CrTe_2$ up to 9T

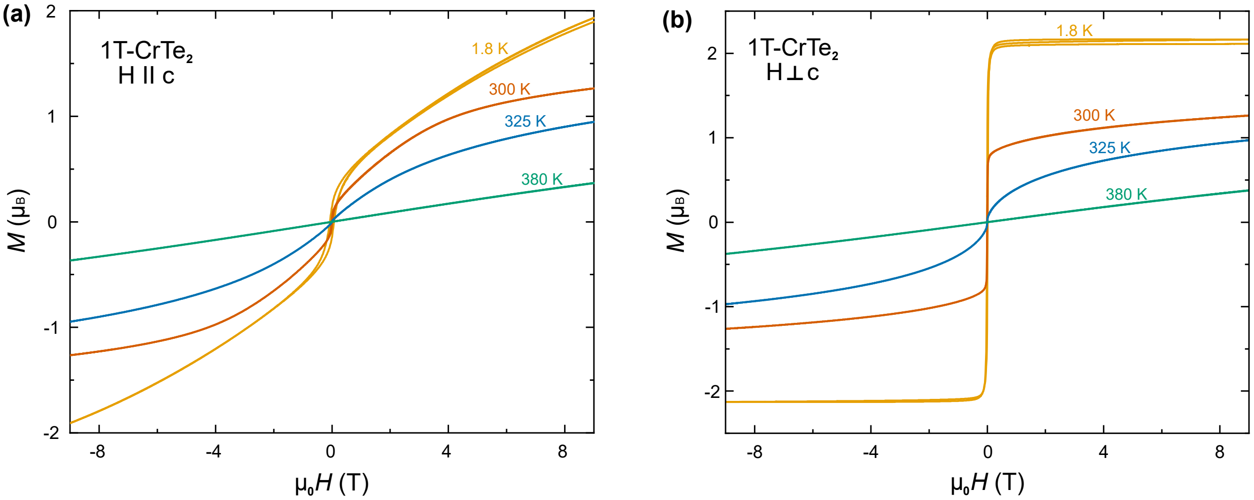

Figure 4: Field-dependent magnetization of 1T-$CrTe_2$ synthesized via $LiCrTe_2$ deintercalation in H_2SO_4, with measurements between -9 T and 9 T along the hard axis (a) and easy axis (b). Here, the hard axis is $H \parallel$ c, and the easy axis is $H \perp$ c.

Magnetic structure of 1T-$CrTe_2$ with magnetic moment contribution in z-direction

Table 1: Summary of possible magnetic space groups (MSG) to describe the magnetic contribution in the neutron powder diffraction of 1T-$CrTe_2$ at $T = 1.6\,K$ based on ISODISTORT from the ISOTROPY software[1,2]

MSG	164.89 P-3m'1
Basis	(1,0,0), (0,1,0), (0,0,1)
Origin	(0,0,0)
MSG	12.58 C2/m.1
Basis	(-1,-2,0), (1,0,0), (0,0,1)
Origin	(0,0,0)
MSG	12.62 C2'/m'
Basis	(2,1,0), (0,1,0), (0,0,1)
Origin	(0,0,0)
MSG	2.4 P-1.1
Basis	(0,1,0), (-1,0,0), (0,0,1)
Origin	(0,0,0)

Table 2: Comparison of the refinement parameters of the magnetic structure of 1T-$CrTe_2$ with and without a magnetic moment contribution in x-direction based on neutron powder diffraction data at $T = 1.6\,K$.

	With m_x	Without m_x
a (Å)	6.5837(14)	6.5829(15)
b (Å)	3.7859(15)	3.7869(17)
c (Å)	6.0264(3)	6.0262(3)
V (Å^3)	75.187(5)	150.22(8)
μ_{Cr} (μ_B)	1.370(14)	1.329(14)
R_p	1.39	1.40
R_{wp}	1.78	1.80
R_{exp}	1.30	1.30
χ^2	1.89	1.92

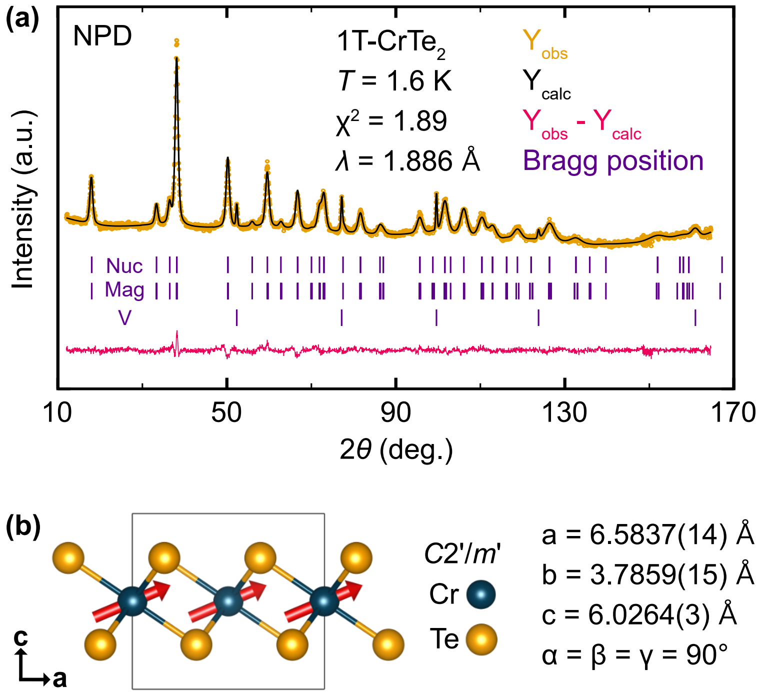

Figure 5: Rietveld refinement of neutron diffraction patterns of H_2SO_4-deintercalated 1T-$CrTe_2$ with a magnetic moment in z direction (a) Refined pattern obtained at $T = 1.6\,\mathrm{K}$ based on three phases: A nuclear part (Nuc) on the basis of SXRD data, a magnetic contribution (Mag) with the space group C2'/m' and the sample contained made of vanadium (COD code: 1506411). (b) Graphical representation of the magnetic structure with parallel orientation of the magnetic moments of Cr represented by red arrows.

DSC and DTA of the thermal composition of 1T-$CrTe_2$ deintercalated using H_2SO_4

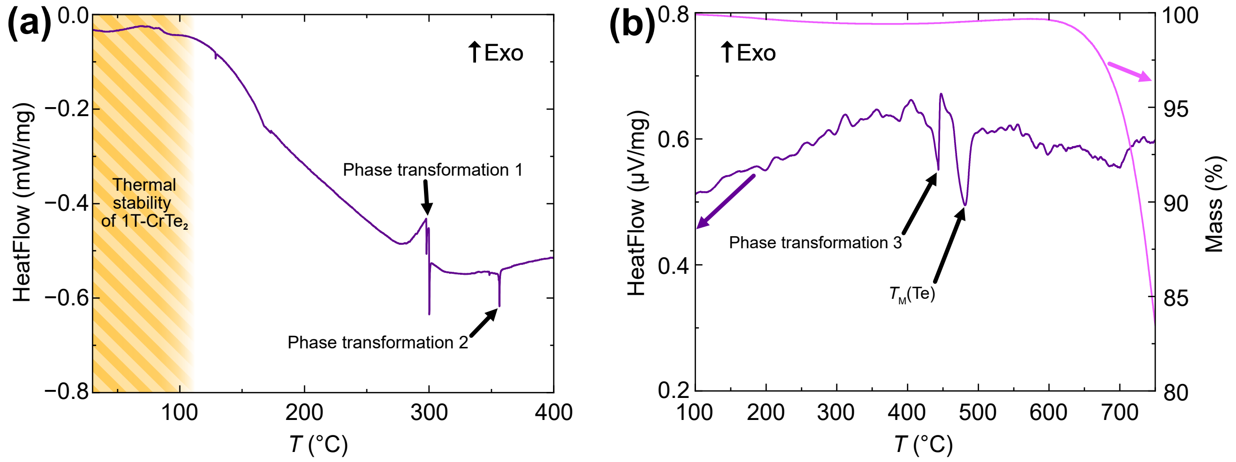

Figure 6: (a) DSC measurement of 1T-$CrTe_2$ synthesized through deintercalation of $LiCrTe_2$ in 1M H_2SO_4, measured between 30 °C and 400 °C. Key thermal events are marked with arrows. (b) DTA/TG measurements of 1T-$CrTe_2$ synthesized similarly. Both were conducted on ground crystals, with exothermic events indicated by positive heat flow in both experiments.

Differential scanning calorimetry (DSC) measurement was performed using a Mettler Toledo DSC1 STARe System. Synthesized 1T-$CrTe_2$ was placed in an aluminum container closed with a lid with a small hole for pressure adjustment and heated with a rate of $5\,K \cdot min^{-1}$ under N_2 atmosphere.

Thermo gravimetry (TG) and Differential thermal analysis (DTA) measurements were performed in a Setaram TAG-24 thermal analyzer. 1T-$CrTe_2$ synthesized using 1M H_2SO_4 was placed in an open alumina Al_2O_3 crucible (without a lid) and was heated at a rate of $5\,K{\cdot}min^{-1}$ from RT to 750 °C under Ar atmosphere.

To investigate the thermal stability and decomposition of acid-assisted deintercalated 1T-$CrTe_2$ under an inert atmosphere, we performed DSC and DTA/TG measurements on acid deintercalated 1T-$CrTe_2$ powders, with these results being presented in Figure 6.

From our DSC measurements, we observe that 1T-$CrTe_2$ is thermally stable up to a temperature of approximately $T \approx 110\,°C$ after which the overall baseline shows a negative

slope, which remains at around -0.5 mW/mg up to around 280 °C at which in the slope becomes positive up about 300 °C. Here the DSC measurement shows two sharp endothermic peaks which were marked as a major phase transition 1. Phase transition 2 was attributed to an endothermic peak with an onset of 355 °C. In the DTA we observed two endothermic signals at about 430 °C and 460 °C the latter of which corresponds well to the melting point of Te around 450 °C. Starting at approximately 600 °C a decrease in mass was detected which is likely the result of the evaporation of Te.

It should be denoted that DSC measurements can be considered more sensitive, which could explain why the DTA does not show the thermal events observed in the DTA. Additionally, the absence of a lid during the DTA measurement might have had an influence as well. Due to the large number of thermal events, which also include two small peaks at 129 °C and 348 °C as well as a change of slope at 165 °C closer investigations should be performed in the future.

Table 3: Exchange couplings of 1T-$CrTe_2$, calculated within GGA+U for the new $T = 120$ K structure. The Cr-Cr distances in the last line identify the exchange path. Errors are statistical uncertainties incurred in the energy mapping procedure.

U (eV)	J_1 (K)	J_2 (K)	J_3 (K)	J_4 (K)	J_5 (K)	J_6 (K)	J_7 (K)	T_{CW} (K)
0.75	-18.2(2.4)	-38.1(2.6)	-21.1(1.8)	-6.3(9)	-10.0(1.6)	2.0(8)	1.8(9)	503
1.	-21.3(2.3)	-36.7(2.4)	-20.1(1.7)	-5.9(8)	-9.5(1.5)	2.1(8)	2.1(9)	499
1.25	-25.1(2.2)	-34.5(2.3)	-18.7(1.6)	-5.7(8)	-8.5(1.4)	2.3(7)	2.4(8)	494
1.5	-29.3(1.9)	-31.9(2.0)	-17.3(1.4)	-5.5(7)	-7.4(1.3)	2.6(7)	2.6(7)	490
1.75	-33.8(1.79	-29.2(1.7)	-15.7(1.2)	-5.3(6)	-6.2(1.1)	2.8(6)	2.8(6)	487
2.	-38.1(1.4)	-26.5(1.5)	-13.9(1.0)	-5.0(5)	-5.1(9)	3.0(5)	2.9(5)	482
2.25	-42.1(1.2)	-23.9(1.2)	-12.3(9)	-4.8(4)	-4.1(8)	3.3(4)	2.9(4)	477
2.5	-45.8(9)	-21.6(1.0)	-10.9(7)	-4.6(4)	-3.3(6)	3.6(3)	2.9(4)	475
d_{Cr-Cr} (Å)	3.7823	6.0203	6.55114	7.10984	7.5646	8.89727	9.66784	

Details on the DFT energy mapping

In Table 3, we present all sets of Heisenberg Hamiltonian couplings that we determined using DFT energy mapping; they are shown graphically in Fig. 8 (a) of the main text. The last line contains the Cr-Cr distances that are used to identify the exchange path. The paths are

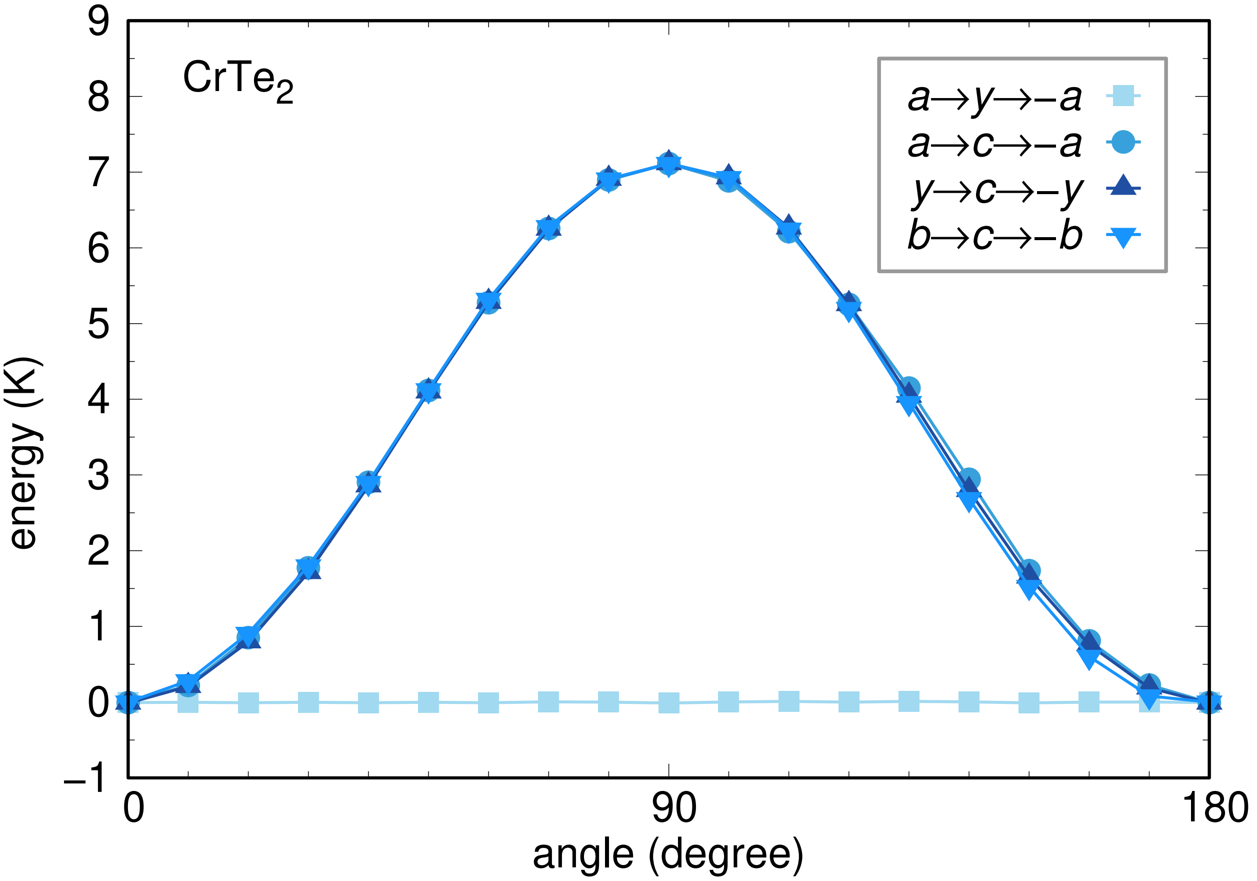

Figure 7: Fully relativistic energies (including spin-orbit coupling) of the ferromagnetic spin configuration as function of the quantization axis. The functional is GGA+U with an on-site energy of $U = 2\,\text{eV}$. a and c axes are parallel to Cartesian x and z axes while b has a 120° angle with a.

shown in Fig. 8 (b) of the main text. The Curie-Weiss temperatures given in the last column of Table 3 are calculated according to

$$\theta_{\rm CW} = -\frac{1}{3}S(S+1)\big(6J_1 + 2J_2 + 6J_3 + 12J_4 + 6J_5 + 12J_6 + 12J_7\big)\,, \tag{1}$$

The ferromagnetic ordering temperature T_C of 1T-CrTe$_2$ depends linearly on the energy scale $\theta_{\rm CW}$ and logarithmically on both the ratio between in-plane exchange interactions $J_\parallel$ and interlayer exchange interactions $J_\perp$ and the single ion anisotropy.[3]

Based on the coordination numbers, we can estimate $J_\parallel \approx J_1 + J_3 + J_5$ and $J_\perp \approx J_2 + 6J_4 + 6J_6 + 6J_7$. Using fully relativistic calculations for the ferromagnetic state that

take the spin orbit coupling into account, we find that both for GGA and GGA+U, the Cr moments are strongly easy plane. In Fig. 7, we show the results for GGA+U with $U = 2\,\mathrm{eV}$. Rotation of the spin configuration in the ab plane yields constant energy while any rotation out of the ab plane increases the energy. Thus, c is the hard axis and ab is the easy plane. Cr moments parallel to c are $12.2\,\mathrm{K}$ per formula unit less favorable than perpendicular to c in GGA, and $7.1\,\mathrm{K}$ per formula unit in GGA+U for $U = 2\,\mathrm{eV}$. If we write the magnetic Hamiltonian as $H = H_{\mathrm{Heisenberg}} + \sum_i K_c (S_i^z)^2$ where $|S^z| = \frac{3}{2}$, we find $K_c = 5.4\,\mathrm{K}$ at the GGA level and $K_c = 3.2\,\mathrm{K}$ at the GGA+U with $U = 2\,\mathrm{eV}$. This makes 1T-$CrTe_2$ strongly easy plane, confirming our experimental observations. The precise determination of the ordering temperature requires classical Monte Carlo simulations of the full Hamiltonian with single ion anisotropy and is beyond the scope of the present study.

References

(1) Stokes, H. T.; Hatch, D. M.; Campbell, B. J. ISODISTORT, ISOTROPY Software Suite, iso.byu.edu.

(2) Stokes, H. T.; Hatch, D. M.; Campbell, B. J. "ISODISPLACE: An Internet Tool for Exploring Structural Distortions.". *Journal of Applied Crystallography* **2006**, *39*, 607.

(3) Yasuda, C.; Todo, S.; Hukushima, K.; Alet, F.; Keller, M.; Troyer, M.; Takayama, H. Néel Temperature of Quasi-Low-Dimensional Heisenberg Antiferromagnets. *Phys. Rev. Lett.* **2005**, *94*, 217201.
